# A phase II multicenter prospective study to evaluate the safety and efficacy of myeloablative-dose treosulfan plus fludarabine (FT14) conditioning regimen in allogeneic hematopoietic stem cell transplantation for AML patients aged 40–65 in first complete remission

**DOI:** 10.1038/s41409-026-02937-7

**Published:** 2026-06-24

**Authors:** Daniele Avenoso, Vera Radici, Alessandro Leoni, Cristina Skert, Massimo Martino, Nicola Mordini, Francesco Saraceni, Attilio Olivieri, Fabio Ciceri, Cristina Tecchio, Alessandra Picardi, Giorgia Saporiti, Francesca Patriarca, Paola Bresciani, Chiara Nozzoli, Denise Maravalle, Nicola Polverelli, Gabriele Magliano, Enrico Morello, Mirko Farina, Simona Bernardi, Federica Re, Luca Garuffo, Simone Maifredi, Silvia Zanon, Rachele De Domenico, Michele Malagola, Domenico Russo

**Affiliations:** 1 Unit of Blood Diseases and Bone Marrow Transplantation, Department of Clinical and Experimental Science, University of Brescia, ASST Spedali Civili di Brescia, Brescia, Italy; 2 Hematology and Bone Marrow Transplant Unit, Ospedale dell’Angelo, Venezia, Italy; 3 Hematology and Stem Cell Transplantation and Cellular Therapies Unit, Grande Ospedale Metropolitano “Bianchi-Melacrino-Morelli”, Reggio Calabria, Italy; 4 Division of hematology, Azienda Ospedaliera S. Croce e Carle, Cuneo, Italy; 5 Hematology and BMT Unit, Ospedali Riuniti di Ancona, Ancona, Italy; 6 Ospedale San Raffaele, Haematology and BMT, Milano, Italy; 7 Department of Engineering for Innovation Medicine, Innovation Biomedicine Section, Hematology and Bone Marrow Transplant Unit, University of Verona, Verona, Italy; 8 Stem Cell Transplant Program AORN Cardarelli, Naples, Italy and Biomedicine and Prevention Department of Tor Vergata University, Rome, Italy; 9 Hematology and BMT Unit, Fondazione IRCCS Ca’ Granda Ospedale Maggiore Policlinico, University of Milan, Milan, Italy; 10 Haematology and Stem Cell Transplantation Unit, Azienda Sanitaria Universitaria Friuli Centrale, DMED, Udine University, Udine, Italy; 11 Hematology Unit and Chair, Azienda Ospedaliera Universitaria di Modena and Department, Modena, Italy; 12 Hematology Department Careggi University Hospital, Florence, Italy; 13 UOC di Ematologia e Terapia Cellulare, Ospedale C e G Mazzoni, Ascoli Piceno, Italy; 14 Unit of Bone Marrow Transplantation and Cellular Therapies, Division of Hematology, Fondazione IRCCS Policlinico San Matteo, Pavia, Italy

**Keywords:** Diseases, Cancer

## Abstract

Evidences supporting the use of myeloablative-dose Treosulfan combined with Fludarabine remain largely retrospective, with outdated prospective studies available. In this phase II multicenter prospective trial, we evaluated the safety and efficacy of FT14 conditioning regimen in patients with acute myeloid leukemia undergoing allogeneic stem cell transplantation in first complete remission.(EudraCT Number:2021-006515-28; Clinical trial number NCT07232953). The study was designed to provide a non-inferior alternative to standard busulfan-containing regimens, which would enable effective transplantation while minimizing toxicity. The primary objective was 1-year Leukemia-free survival (LFS). In total, 82 patients were enrolled in the study with a median follow up of 19.7 months; donor used were matched sibling in 22, matched unrelated 52 and mismatched unrelated in 8 cases, respectively. LFS at 180-day and 365-day were 87.8% and 81.7%, respectively. Cumulative incidence of relapse at 1 year after allo-HSCT was 14.9% with mean time to relapse of 5.6 months. Also, FT14 regimen provided an excellent safety with nearly absent NRM, no cases of septic death, and no cases of primary graft failure. Our study supports the use of FT14 as effective myeloablative conditioning regimen for AML patients aged 40–65 y in first complete remission, especially in patients for whom busulfan based conditioning regimens poses excessive toxic risks.

## Introduction

In the last two decades, the use of allo-HSCT progressively increased, and currently, it is offered to intermediate-high risk AML and in first complete remission (CR1) without a specific age limitation [1], since age limit moved on till 75 years old. Expanding transplant benefits to a broader population of AML patients [2–4] coincided with advancements in safer and more effective transplant procedures [5], including improved recipient and donor selection, enhanced prophylaxis and treatment of GvHD and infections [6–11], and refined management of the myelosuppressive and immunosuppressive effects associated with newer and lesser toxic conditioning regimens. A recent study conducted by GITMO on two thousand AML patients who underwent allo-HSCT in Italy over the past two decades revealed significant changes in the conditioning regimens used during this period [12, 13]. The most notable changes included the abandonment of total body irradiation (TBI) and cyclophosphamide, the widespread adoption of intravenous busulfan-based platforms, a gradual reduction in the use of Myeloablative Conditioning (MAC) regimens, and, moreover, a shift from RIC regimens to Reduced-Toxicity myeloablative Conditioning (RTC) regimens aimed at reducing transplant-related toxicity [13] without compromising anti-leukemic activity.

Currently, the combination fludarabine and busulfan (FB4) is considered the gold-standard myeloablative regimen for AML patients aged 40–65 undergoing allo-HSCT. As reported by Rambaldi et al., in AML patients, the FB4 conditioning regimen demonstrated a significantly lower non-relapse mortality (NRM) with the same anti-leukemic activity compared to the busulfan plus cyclophosphamide (BuCy) regimen [14].

However, improving allo-HSCT conditioning regimens with highly anti-leukemic activity and low host toxicity remains an unmet medical need, considered the increasing number of AML patients older than 60 years, even with comorbidity, now deemed fit for allo-HSCT [15].

In this view, treosulfan showed to be an interesting agent in conditioning regimens for allo-HSCT for AML. Compared to traditional agents like busulfan, treosulfan showed to have a high antileukemic activity, a low organ toxicity and reduced risk of GvHD and NRM [13, 16].

Due to its peculiar profile, it was used in combination with Fludarabine in old and frail patients with AML or MDS with comorbidities and occasionally with advanced disease or for second transplant procedures [16]. A reduced dose of Treosulfan (10 g/m^2^) in combination with Fludarabine (FT10) has been registered by EMA and other Medical Regulatory Authorities for elderly or frail AML and MDS patients eligible for allo-HSCT [13]. This is consequence of a prospective randomized study where the superiority of FT10 over FB2 was evident in terms of NRM. Nowadays, it is considered the gold standard conditioning regimen for frail patients with AML or MDS older than 60 years [13].

Clinical experiences on the use of myeloablative doses of Treosulfan i.e., 14 g/m^2^ in combination with Fludarabine are mainly retrospective [17–19] and those prospective very limited and old dated. Herein we report the results from a multicenter, prospective clinical study of patients with AML aged 40–65 years who underwent allo-HSCT following myeloablative conditioning with fludarabine (30 mg/m^2^ for five days) and treosulfan (14 g/m^2^ for three days), with the aim to acquire information on its safety and efficacy, in view of a potential comparison with the current standard FB4 conditioning regimen

## Patients and methods

From September 2022 to December 2024, 82 patients have been enrolled in the study by 13 Italian GITMO (Gruppo Italiano Trapianto Midollo Osseo) Transplant Programs. AML was diagnosed according to WHO 2016 classification and stratified according to ELN 2017 criteria [20, 21]. The study was designed as a prospective multicentric single arm phase II study with the aim to measure the 1-year LFS after FT14 with HLA identical donor (both sibling and unrelated) conditioning in AML patients aged 40–65 yy and in first complete remission according to European Leukemia Network criteria of 2017 [21]. Eligible patients were aged >40 and <65 years and had a diagnosis of AML in first complete remission (CR, CRi, or MLFS).

Patients with an HLA-matched related donor or matched (10/10) or single-locus mismatched unrelated donor (9/10) were eligible.

Adequate organ function was required, including hepatic function (bilirubin ≤2× upper normal limit and ALT/AST ≤ 2.5× upper normal limit), renal function (creatinine clearance ≥50 mL/min), and an ECOG performance status <2. Patients had to be willing and able to comply with all study requirements and provide written informed consent. Exclusion criteria included AML with t(15;17), t(8;21) or inv(16) without additional adverse cytogenetic abnormalities or negative MRD, known active CNS involvement, grade >2 NCI-CTCAE v5 adverse events at enrollment, significant organ dysfunction (left ventricular ejection fraction <40%; FEV1, FVC or DLCO < 40% of predicted; liver function tests >5× upper normal limit; creatinine clearance <40 mL/min), evidence of active HBV or HCV infection (HBV-DNA or HCV-RNA positivity), HIV infection, current uncontrolled infections, other life-threatening comorbid diseases, hypersensitivity to study medications, or participation in another clinical trial within 30 days prior to study start. The study was conducted in accordance with the Declaration of Helsinki and Good Clinical Practice guidelines. The study protocol was approved by the Ethics Committee of ASST Spedali Civili di Brescia, Italy, and authorized by the Italian Medicines Agency (AIFA; authorization no. 0038756 of 31 March 2022; EudraCT number 2021-006515-28). Written informed consent was obtained from all participants prior to enrollment

### HLA tissue typing and matching

Donors and recipients were typed using Third Generation Sequencing (TGS) and Next Generation Sequencing (NGS) techniques for HLA-A, -B, -C, -DRB1, -DQ at a high-resolution level. Donors were considered fully matched when a 10/10 allele-level match was present, while single-locus mismatched unrelated donors were defined as 9/10 matches.

### Measurable residual disease (MRD)

MRD was assessed in all patients before transplantation. MRD evaluation was performed using multiparametric flow cytometry (MFC) in cases with an identifiable leukemia-associated immunophenotype and/or by molecular techniques (RQ-PCR or digital PCR) for patients with trackable molecular markers (e.g., NPM1, RUNX1-RUNX1T1, CBFB-MYH11, FLT3-ITD). A threshold of 0.1% for flow cytometry and a sensitivity of at least 10⁻⁴ for molecular assays were considered. MRD was categorized as positive or negative according to local accredited laboratory standards [22].

### HSCT procedures

HSCT was performed with G-CSF-mobilized peripheral blood stem cells (PBSC) or following bone marrow harvest under general anesthesia. Conditioning protocol was administered using IV fludarabine 30 mg/m^2^ on days –6, –5, –4, –3, –2 and IV treosulfan 14 g/ m^2^ on days –4, –3, –2 (FT14). GvHD prophylaxis consisted of in-vivo T-cell depletion with Anti-Thymocyte globulin (ATG)-Grafalon (Grafalon, Neovii Biotech GmbH, Munich, Germany) or ATG-Thymoglobuline (Thymoglobuline, Sanofi-Genzyme, Cambridge, MA). ATG-grafalon was given at 10 mg/kg/day (from day –4 to day –2) in patients receiving transplant from Matched Unrelated Donor (MUD) and at 5 mg/kg/day (from day -4 to day -2) in patients receiving transplant from Matched Sibling Donor (MSD). Alternatively, ATG-Thymoglobuline was used at the following dosages: 2,5 mg/kg/day (day -3 and -2) in MUD and 2,5 mg/kg/day (day –2 only) in MSD, respectively. Methotrexate 15 mg/m^2^ on day +1, 10 mg/ m^2^ on days +3 and +6, and ciclosporin 3 mg/kg from day -1 (therapeutic level of 150–200 µg/L) until day +56, which was then tapered in absence of GvHD until discontinuation on day +90.

Neutrophil engraftment was defined as neutrophil counts ≥500/μL for three consecutive days without Granulocyte colony-stimulating factor (G-CSF) support. Platelet engraftment was defined as platelet count ≥20,000/μL for three consecutive days or ≥50,000/μL without receipt of platelet transfusions in the two previous days.

Chimerism analysis of peripheral blood and bone marrow was performed on days +28, +100, +180, and +365 with short tandem repeat (STR) testing by polymerase chain reaction (PCR) followed by fragment analysis as previously described [23].

Graft failure was defined as Primary if <5% donor chimerism at day +28. Secondary graft failure (graft rejection) is defined as initial recovery followed by neutropenia with <5% donor chimerism.

### Antinfective prophylaxis and treatment

Anti-bacterial, anti-fungal, and anti-viral prophylaxis will be administered according to local anti-infective policy based on international guidelines [7, 24]. Anti-Citomegalovirus (CMV) prophylaxis with letermovir (Prevymis, Merck) was administered to all CMV IgG-positive patients [25] within 7 days from transplant as per prescribing information. Bi-weekly CMV, adenovirus, and EBV monitoring by PCR begun at time of conditioning, until day +100, followed by weekly testing until day +180. Patients received red blood cell and platelet transfusions according to institutional protocols.

### Diagnosis and treatment of GVHD and veno-occlusive disease/sinusoidal-occlusive disease (VOD/SOS)

The presence of acute and chronic GvHD (aGvHD and cGvHD, respectively) was determined clinically, based on standard criteria (Glucksberg grading system for aGvHD and NIH criteria for cGvHD) [26], and confirmed, where possible, by histologic analysis of skin and/or gastrointestinal biopsy specimens. First- and second-line therapy for GvHD was provided according to institutional protocols based on international consensus and guidelines. Veno-occlusive disease/sinusoidal-occlusive disease (VOD/SOS) was diagnosed as per previous definition [27] and monitored as previously described [28].

### Sample size and study population

The study was originally designed as a non-inferiority trial, assuming a lower toxicity of the FT14 compared to fludarabine and myeloablative dose of busulfan (FB4 conditioning). The sample size required was calculated based on the of lower toxicity and higher efficacy of FT14 compared to fludarabine and myeloablative dose of busulfan (FB4 conditioning). A sample size of 82 patients achieves a power of 80% to detect a non-inferiority of 5% using a one-sided exact one binomial test with a significance level alpha of 0.05. These results assume a 1-year LFS of 65% (FB4) and the difference in the 1-year LFS under the alternative hypothesis is +8%. Null hypothesis will be rejected if 57 or more patients will be alive and disease-free at 1 y after transplant. The assumed 1-year LFS of 65% was derived from the Busulfan-Fludarabine arm of the randomized GITMO trial by Rambaldi et al. [14], which represents the current benchmark myeloablative conditioning regimen in this age group. If ≥61 patients will be alive and leukemia-free at 1 year, FT14 superiority over the historical FB4 benchmark will be suggested.

### Statistical analysis and study objectives

Continuous variables were described as mean and standard deviation (or median and IQR, if non-normally distributed), and categorical variables as frequencies.

Overall survival (OS) was estimated using the Kaplan-Meier curves, with patients alive being censored at the time of last follow-up; OS was defined as time from HSCT to death from any cause or the last follow-up for living patients. Leukemia-free survival (LFS) was estimated with Kaplan-Meier curves and defined as time from HSCT to relapse or death (whichever came first) or last follow-up, with patients alive or without events being censored at the time of last follow-up. GvHD-relapse free survival (GRFS) was defined as time from HSCT to either acute grade 3/4 GvHD, moderate-severe chronic GvHD, relapse or death from any cause. Cumulative incidence analysis with competing risks was performed for NRM, relapse, and GvHD (either acute or chronic). The Fine and Gray distribution hazard model was used to estimate the cumulative incidence functions, considering non-relapse mortality as a competing risk for relapse, and relapse as a competing risk for NRM. NRM was defined as death due to any cause other than progression of the underlying malignancy, with death due to relapse as a competing event. Relapse was defined as recurrence of the underlying hematological malignancy; death due to any other cause (NRM) was a competing event for this analysis. For GvHD cumulative incidence analysis, death without aGvHD in first 100 days was considered a competing event for aGvHD endpoint, whereas relapse or death in absence of cGvHD were considered as competing events for cGvHD outcome. The multivariate model was built following the same rules as for the Cox model. For the Cox model, hazard ratios (HRs) are reported. Chi square statistics was used for comparison of categorical variables while continuous variables were compared with Mann–Whitney test for non-normally distributed data, and Student’s *t* test for normally distributed data.

The primary objective was 1-year LFS after FT14 conditioning in AML patients in complete morphological remission (CR/CRi/MLFS) aged 40–65 y.

Secondary objectives were:

- Cumulative incidence of graft failure at +30 days and +100 days from transplant
- NRM at +30 days and +100 days from allo-HSCT
- Cumulative incidence and severity of acute GvHD at 100 days after transplant
- Cumulative incidence and severity of chronic GvHD at 1 year after transplant
- Overall Survival (OS) at 1 year after transplant
- Relapse Incidence (RI) at 6 months and 12 months after transplant
- 1-year probabilities of GvHD-free/relapse-free survival (GRFS)
- Safety of FT14 regimen in terms of conditioning-related adverse events

The primary endpoint was assessed when the last enrolled patient reached 12 months of follow-up. All analyses were done with RStudio software (version 4.3.2), and two-tailed *p* value was considered significant when ≤0.05.

## Results

### Patients’ characteristics

The median age at the time of allo-HSCT was 56 years (interquartile range [IQR], 51–60.75). The median time from diagnosis to allo-HSCT was 165 days (IQR, 133–217). Although the protocol allowed the inclusion of patients in CR, CRi, or MLFS according to ELN criteria, all patients included in the present cohort were transplanted in classical complete remission with full hematologic recovery. All screened patients who were subsequently enrolled underwent allo-HSCT. The database was updated for the present analysis when all enrolled patients had reached at least 12 months of follow-up after transplantation. At the time of the updated analysis, the median follow-up was 19.7 months (range 16–25). Patients’ baseline characteristics and transplant outcomes are summarized in Tables 1 and 2. Unrelated donors were in 73% of cases, while 27% of patients received grafts from sibling donors. According to the 2016 WHO criteria: 31 patients (38%) had an AML with recurrent genetic abnormalities, 26 (32%) AML with myelodysplasia-related changes, 20 (24%) AML not otherwise specified (NOS) and five (6%) had a therapy-related myeloid neoplasm. According to the 2017 ELN risk stratification, most patients had either intermediate (41%) or adverse (48%) risk. All the patients with favorable ELN risk (*n* = 9, 11%) were included in the trial due to a measurable residual disease (MRD) positivity post-consolidation chemotherapy. In addition, 8 patients were NPM1 positive with normal karyotype and 1 case of CBFB::MYH11 with positive MRD. Based on cytogenetic risk criteria, 21% of patients had an abnormal karyotype, including 6 patients (7% of the total cohort) with a complex karyotype. Measurable residual disease (MRD) status at the time of transplantation was performed for 82 out of 82 patients (100%). Among them, 21 patients (26%) underwent allo-HSCT with a positive MRD status: 6 patients were positive by flow cytometry, 14 by molecular MRD testing, 1 by cytogenetic analysis. At the time of analysis, the median follow-up was 19.7 months (range 16–25 months).

**Table 1:** Patients characteristics.

Variable	Total	%
Patients evaluable	82	100%
Median age (y) (IQR)	56 (51–60.75)	–
**Ethinicity**		
Hispanic or Latino	4	5%
Caucasian	78	95%
**Diagnosis according to WHO 2016**		
AML with recurrent genetic abnormalities	31	38%
AML with myelodysplasia-related changes	26	32%
AML, NOS	20	24%
Therapy related myeloid neoplasm	5	6%
**ELN 2017 risk stratification**		
Favorable	9	11%
Intermediate	34	41%
Adverse	39	48%
**Performance status**		
ECOG 0	61	74%
ECOG 1	21	26%
**Karyotype**		
Normal	51	62%
Abnormal	17	21%
Not done for medical reasons	14	17%
**Molecular Biology (RT-qPCR, Nested-PCR, or HPLC)**		
NPM1	15	18%
FLT3-ITD	6	7%
FLT3-TKD	2	2%
**Donor type**		
Sibling	22	27%
Matched unrelated	52.	63%
Mismatched unrelated	8.	10%

**Table 2:** Toxicities and complications.

Adverse event	*N* (%)
**Acute GvHD**	
Grade I	15 (18)
Grade II	6 (7)
Grade III-IV	4 (5)
**Chronic GvHD**	
Mild	5 (6)
Moderate	1 (1)
Severe	0
**Mucositis**	
Grade 1	9 (11)
Grade 2	12 (15)
Grade 3	2 (2)
Grade 4	0
**VOD / SOS**	0
**Infections**	24 (29)
**Fatal infections**	0
**Relapse within day 100**	2 (2)
**Relapse after day 100 and within day 180**	6 (7)
**Relapse after day 180 and within day 365**	3 (3)
**Relapse after day 365 and within day 720**	1 (1)

### Graft characteristic, engraftment and chimerism

The main source of the graft was unmanipulated peripheral blood hematopoietic stem cells (95%); only three patients received a bone marrow transplant. A median of 6.8 × 10⁶ CD34⁺ cells/kg (IQR, 5.02–15 × 10⁶) and a mean of 148 × 10⁶ CD3⁺ cells/kg (range, 48.8–239) were infused. All patients (100%) achieved hematologic recovery. The median time to achieve a neutrophil count ≥1000/μL was 17days (IQR, 13–21), and 17 days (IQR, 14–21) for platelet recovery to ≥50,000/μL. No deaths occurred before engraftment, and no cases of graft failure were observed.

### Leukemia-free survival

**Fig. 1:**
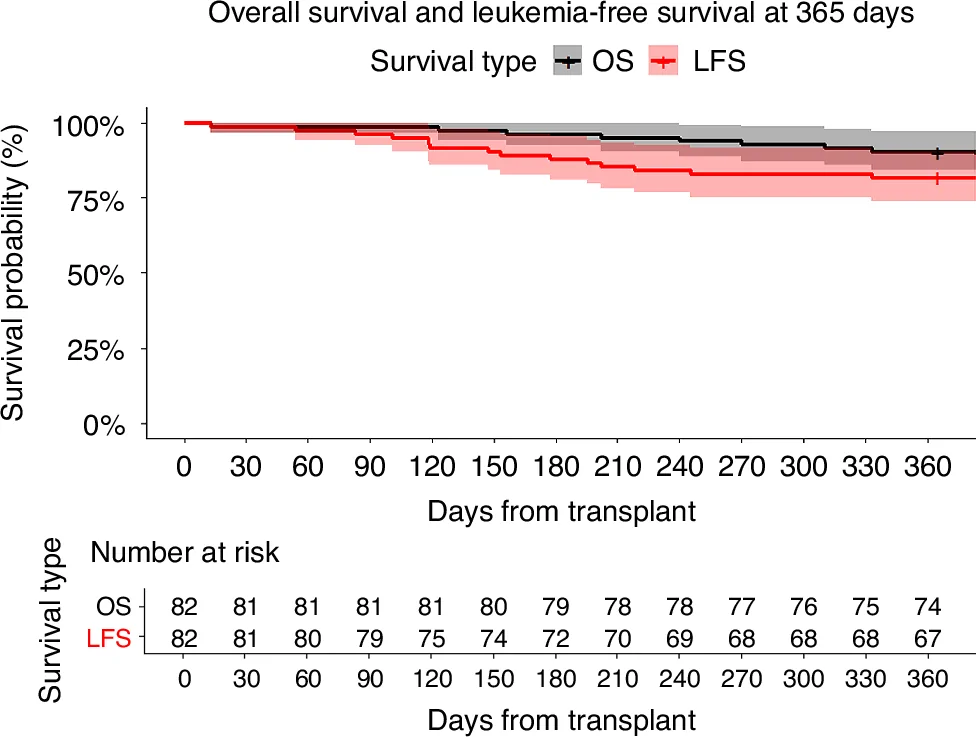
Overall survival and leukemia-free survival at one year for the FT14 population.

Leukemia-free survival was 87.8% at 6 months (95% CI 81-95.2) and 81.7% at 1 year (95% CI: 73.8–90.5%; Fig. 1—red curve), with median LFS not reached. At 1 year, 67 patients were alive and disease-free, exceeding the predefined threshold of 61 required by the statistical design; therefore, the primary endpoint was met. Although the study was not designed as a randomized comparison, these findings suggest that FT14 may represent an effective alternative to FB4-based conditioning and warrant further evaluation in comparative studies.

### Overall survival, GVHD-relapse-free survival, and relapse rate

One-year OS was 90.2%, with median OS not reached (Fig. 1—black curve).

The probability of GRFS at 6 months and one year after transplant were 81.73% and 74.4%, respectively. The median GRFS time was not reached during the observation period.

**Fig. 2:**
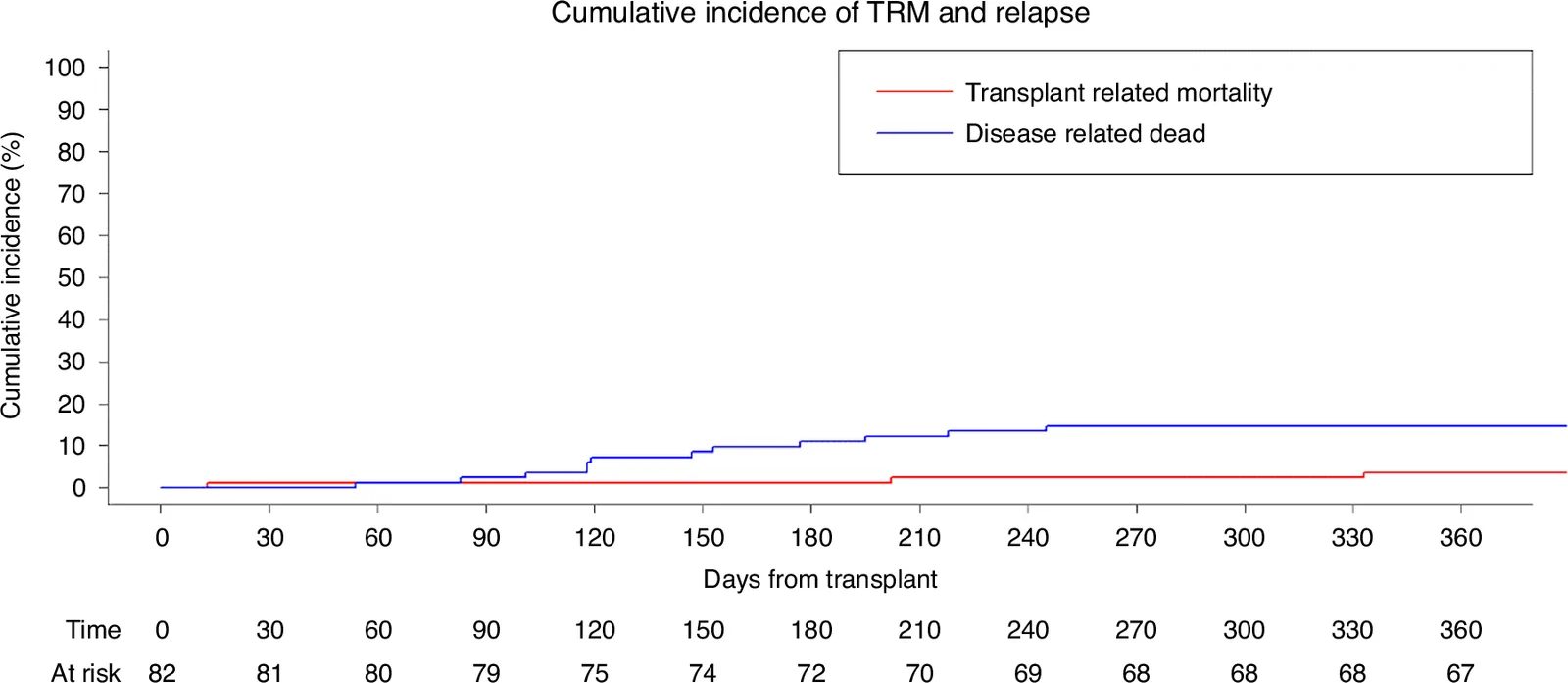
Cumulative incidence of transplant-related mortality and relapse.

Relapse occurred in twelve patients (15%) with a mean time to relapse of 168.2 days (range 54–392). In relation to these patients, two relapsed in the first three months post-transplant, 6 in the first six months, 3 between six months and one year, and only 1 after one year. The cumulative incidence of relapse was 10.30% at 6 months and 14.9% at 1 year. Measurable residual disease (MRD) status at time of transplant also did not influence the relapse rate in this cohort (*p* value not significant—Log-rank). Nine out of 82 patients died (11%), with 6 out of 82 (7%) due to disease progression and 3 out of 82 (4%) due to multiorgan failure (2 out of 3) and severe aGvHD (1 out of 3). The NRM at day +180 and +365 days was 1.21% and 3.7%, respectively (Fig. 2). No cases of infective deaths were recorded in this population.

### Graft versus host disease

According to Glucksberg criteria, aGvHD occurred in 30% of cases. Grade I aGvVHD was observed in 15 patients (18%) but it did not require any treatment. Grade II-IV aGvHD occurred in 10 out of 82 patients (12%), including 1 case (1%) of grade IV.

Median time from transplant to aGvHD onset was 29 days (IQR: 19–42.5). There have been six cases of chronic GvHD (7%), mainly mild to moderate and the median time from transplant to cGvHD was 127 days (IQR:110.2–170). The cumulative incidence of chronic GvHD was 5.16% at 6 months and 6.57% at 1 year after allo-HSCT.

### Adverse events and complications

Infectious complications were present in 24 patients (29%), none of which were fatal. Four patients (5%) experienced CMV reactivation without evidence of any organ involvement. 8 patients (10%) showed Epstein-Barr (EBV) viremia, with no cases of posttransplant lymphoproliferative disease (PTLD). Five patients (6%) had a positive test for SaRS-CoV2 and only one had a HHV6 reactivation. Mucositis incidence following transplant was low: only 23/82 patients (28%) experienced oral or gastrointestinal mucositis. Based on WHO grading, 9 cases (11%) were grade 1, 12 cases (15%) grade 2, 2 cases grade 3 (2%), and no grade 4 cases were recorded. Notably, no cases of veno-occlusive disease (VOD) were reported.

## Discussion

This study prospectively investigated the safety and efficacy of the FT14 conditioning regimen in AML patients aged 40–65 years undergoing allo-HSCT in first complete remission (CR1). The study was designed to assess whether FT14 could represent a non-inferior alternative to standard busulfan-containing regimen (FB4), enabling effective myeloablation while minimizing toxicity. However, it should be emphasized that the comparison with the FB4 regimen reported by Rambaldi et al. is indirect, as our study was designed as a single-arm trial using a historical benchmark.

Our results show that FT14 provided excellent safety, with a nearly absent transplant-related mortality (NRM), and no cases of septic death or primary graft failure. This is remarkable considering the inclusion of patients up to 65 years of age, a population typically less tolerant of myeloablative conditioning. Moreover, the one-year leukemia-free survival (LFS) and overall survival (OS) rates strongly support the anti-leukemic efficacy of FT14, suggesting that this regimen may represent a viable alternative to FB4, particularly in patients with high-risk disease. At one-year post-transplant, 81% of patients (67 out of 82) were alive and relapse-free, suggesting not only non-inferiority but also a potential superiority of FT14 over FB4, warranting further investigation. Treosulfan, with its distinct toxicity profile, offers a myeloablative strategy associated with reduced organ toxicity. Previous studies have shown a lower risk of organ toxicity and NRM compared to busulfan [13, 17]. Compared with prior studies establishing the FB4 combination as the standard regimen [14], FT14 demonstrated comparable efficacy while reducing adverse effects, particularly gastrointestinal mucositis, hepatic, and pulmonary toxicity. The reduced mucosal injury likely explains the lower incidence of bloodstream infections and acute GVHD observed. This decrease in toxicity is crucial for extending transplant eligibility to older or more comorbid patients, addressing an unmet clinical need. A recent study by Shimoni et al. (2018) comparing busulfan and treosulfan confirmed that treosulfan significantly reduces the risk of severe complications such as hepatic veno-occlusive disease (VOD), a well-known adverse event associated with busulfan [29]. The absence of VOD in our cohort further supports FT14 as a very good myeloablative conditioning option for AML. Using the same GVHD prophylaxis protocol as Rambaldi et al. [14], another relevant finding was the low incidence of both acute and chronic GVHD. Grade II–IV acute GVHD occurred in 10 patients (12%), while chronic GVHD developed in only 6 patients (8.5%). This favorable GVHD profile, together with the low relapse rate, resulted in a high one-year GRFS, which is very encouraging compared with current standards.

These findings suggest that CsA–MTX–ATG–based GVHD prophylaxis, as used in the FB4 GITMO study [14], combined with treosulfan, may reduce transplant-related toxicity and supports the potential role of ATG-based prophylaxis as alternative to PTCy.

In conclusion, our study supports the use of fludarabine combined with myeloablative-dose treosulfan as a safe and effective conditioning regimen for AML, particularly in patients for whom is expected that busulfan poses excessive toxicity risks. The results are highly exciting in terms of safety and tolerability. Longer follow-up is required to confirm the durability of anti-leukemic activity, even it has to be underlined that most relapses occurred within the first six months, with only one at one year and one thereafter. Looking forward, randomized studies directly comparing FT14 with busulfan-based regimens—mainly FB4—are warranted to further validate the advantages of treosulfan and provide clinicians with better-tailored conditioning strategies for AML patients with limited tolerance to standard busulfan regimens.

## Data Availability

The data that support the findings of this study are available from the corresponding author upon reasonable request. The data are not publicly available because they contain information that could compromise the privacy of research participants.
